# Interpretable machine learning models to predict survival in esophageal cancer: a study based on the SEER database and external validation in China

**DOI:** 10.3389/fphys.2025.1665383

**Published:** 2025-10-29

**Authors:** Abudouresuli Tuersun, Saimaitikari Abudoubari, Abudoushalamu Abudouwake, Huerxidan Tuerdi, Abulizi Maimaitiyiming, Pahatijiang Nijiati, Ya Qiu, Jianquan Wang

**Affiliations:** ^1^ Department of Radiology, The First People’s Hospital of Kashi Prefecture, Kashgar Prefecture, China; ^2^ Xinjiang Key Laboratory of Artificial Intelligence Assisted Imaging Diagnose, The First People’s Hospital of Kashi Prefecture, Kashgar Prefecture, China; ^3^ Shanghai Key Laboratory of Artificial Intelligence, Shanghai, China; ^4^ Department of Dermatology, The First People’s Hospital of Kashi Prefecture, Zhoukou, China; ^5^ Department of Geriatrics, Shache County People’s Hospital, Zhoukou, China; ^6^ Department of Ultrasound, Shache County People’s Hospital, Zhoukou, China; ^7^ Remote Information Center, The First People’s Hospital of Kashi Prefecture, Kashgar Prefecture, China

**Keywords:** esophageal cancer, interpretable machine learning, overall survival, SurvSHAP, prediction model

## Abstract

**Objective:**

We developed interpretable machine learning (ML) models to predict the overall survival (OS) of esophageal cancer patients. This approach aims to make our modeling results more interpretable and transparent.

**Methods:**

We collected the clinicopathological information of esophageal cancer patients from the SEER database and divided them into training and validation sets at a ratio of 7:3. Meanwhile, we obtained an external validation cohort from the First People’s Hospital of Kashi in Xinjiang, China. Using LASSO and multivariate Cox regression analyses, we identified relevant risk factors and combined them to develop CoxPH and 6 ML models: Random Survival Forest (RSF), Gradient Boosting with Component Linear (GLMboost), decision tree (dt), boosting tree (bt), DeepSurv, and neural multi-task logistic regression (NMTLR). We evaluated the predictive performance of these ML models using the C-index, integral cumulative/dynamic AUC, integral Brier score, Kolmogorov-Smirnov (KS) test and Cramer-von Mises (CvM) test. For interpretability assessment, we employed three complementary methods: (1) time-dependent variable importance to quantify feature contribution across follow-up periods; (2) partial correlation survival plots to visualize individual variable effects; and (3) aggregated survival SHapley additive interpretation (SurvSHAP) plots with mean absolute deviation metrics to validate feature impact stability at both individual and population levels.

**Results:**

The final ML model consisted of 11 factors: grade, stage, T stage, N stage, M stage, radiotherapy, chemotherapy, bone metastasis, liver metastasis, lung metastasis, and age. Our predictive models demonstrate significant discriminative power; in particular, the NMTLR model performs best. For the training, validation, and external validation sets, the area under the curve (AUC) for one-, three-, and 5-year OS was higher than 0.81, and the integrated Brier score was consistently lower than 0.175. interpretability analyses confirmed consistent predictive logic: M stage, N stage, age, grade, bone metastases, liver metastases, lung metastases and radiotherapy were identified as the most influential predictors via quantifiable SurvSHAP values and time-dependent importance weights, with their effects visually validated through partial correlation survival curves.

**Conclusion:**

The NMTLR prognostic model is the most effective at predicting the OS of esophageal cancer patients. It helps physicians correctly assess patient survival and provides valuable information for diagnosis and prognosis evaluation.

## 1 Introduction

Oesophageal cancer is a significant malignant tumour of the gastrointestinal tract, occurring between the hypopharynx and the oesophagogastric junction. It ranks as the ninth most common malignancy worldwide, with notably high incidence and mortality rates: in 2020, its global incidence was 3.1% and mortality 5.5%, with 600,000 new cases reported in 2021 alone (Sung et al., 2021; Fernández-Montes et al., 2022). Despite advances in diagnosis and treatment, OS remains unsatisfactory, with a low 5-year survival rate. Thus, accurate survival prediction, personalised treatment planning, and improvements in survival quality remain critical priorities in oesophageal cancer research (Zhang et al., 2020; Helminen et al., 2023).

In recent years, ML has emerged as a powerful tool for prognostic assessment in oncology, including oesophageal cancer. Compared with traditional statistical methods, ML algorithms excel at mining complex patterns from large clinical datasets, enabling more accurate predictions (Li et al., 2024; Sharma and Hassan, 2022). For example, ML models have been developed to predict survival in gastric cancer using genetic algorithm-based Cox regression (Xin et al., 2020), and multi-task logistic regression and random forest models have been applied to estimate tumour survival in oesophageal cancer patients (Jung et al., 2023). However, a key limitation of existing ML-based prognostic models—including those for oesophageal cancer—is their lack of interpretability. Many models function as “black boxes,” where the logic behind predictions and the critical factors driving outcomes remain obscure. This opacity hinders clinicians’ ability to trust or act on model outputs, limiting their translational value in guiding comprehensive treatment strategies (Nayarisseri et al., 2021; Bernard et al., 2023).

The importance of interpretability in clinical ML models cannot be overstated. For prognostic tools to effectively support decision-making, clinicians must understand how and why a model generates a specific prediction—e.g., which patient characteristics (e.g., tumour stage, metastasis status) most strongly influence survival estimates. Such transparency not only enhances trust but also enables validation of model logic against clinical knowledge, ensuring predictions align with biological and pathological mechanisms (Li et al., 2024; Bernard et al., 2023). Despite this need, interpretable prognostic models specifically for oesophageal cancer remain underdeveloped, leaving a critical gap in the field.

To address this gap, our study aimed to develop interpretable ML models for predicting OS in oesophageal cancer patients. We collected clinicopathological data from the SEER database (split into training and validation sets) and an external validation cohort from the First People’s Hospital of Kashi, China. Using LASSO and multivariate Cox regression to screen key risk factors, we constructed 7 ML models (including CoxPH, Random Survival Forest, and Neural Multi-Task Logistic Regression). Beyond evaluating predictive performance, we employed multiple interpretive tools to clarify the models’ decision logic. By integrating accuracy with transparency, we seek to provide a reliable foundation for personalised diagnosis and prognosis in oesophageal cancer.

## 2 Materials and methods

### 2.1 Data sources

In this study, we analysed the clinical data of patients diagnosed with oesophageal cancer between 2010 and 2017. Patient records were identified using International Classification of Diseases for Oncology, Third Edition (ICD-O-3) codes (8850–8858) within the SEER programme. The SEER study data could be accessed easily using the SEER*Stat 8.4.1.2 software, which is available to download from the official website (https://seer.cancer.gov/seerstat/download/). Exclusion criteria were implemented to ensure data quality, including the following: (1) lack of information on survival months, (2) missing or incomplete American Joint Committee on Cancer (AJCC) tumour staging data (T, N and M stages), (3) unknown histological grading information, and (4) incomplete information on the SEER integrated summary stage.

According to the specified inclusion and exclusion criteria, a total of 5,702 patients were recruited into this study. These patients were randomised into two groups: a training group (n = 3,991) and a validation group (n = 1,711), maintaining a ratio of 7:3. Additionally, clinical data for the external validation set (n = 300) were obtained from patients treated at the First People’s Hospital of the Kashi Region between 2015 and 2020 (Approved by the Ethics Committee of the First People’s Hospital of Kashi Region). Throughout the study period, three independent investigators performed data collection: two extracted the data and the third ensured its accuracy. Notably, all patient data were anonymised and no informed consent form was required.

### 2.2 Variable set

In the present study, various clinical parameters were collected, including age at diagnosis, gender, grade, primary site (lower third of oesophagus, middle third of oesophagus, upper third of oesophagus), histology (adenomas and adenocarcinomas; squamous cell neoplasms; epithelial neoplasms; cystic, mucinous and serous neoplasms), Stage (Localised; Regional; Distant), AJCC 7th TNM stage, tumour metastasis (bone, liver, and lung metastasis), and treatment modality (radiotherapy and chemotherapy). The primary outcome metric used in this study was OS, defined as the time interval between the date of diagnosis and the occurrence of death or the most recent follow-up.

### 2.3 Determining prognostic factors for survival

All variables in this study were converted to categorical variables and expressed as frequencies and proportions. To mitigate overfitting, the Least Absolute Shrinkage and Selection Operator (LASSO) method was primarily used to select relevant predictive features. Important prognostic factors were then identified using multivariate analysis and the Cox proportional risk model. Corresponding 95% confidence intervals (CIs) were calculated for all potential risk factors.

### 2.4 Model development

This study used traditional COX regression model, four ensemble learning models (RSF, GLMboost, decision tree, boosting tree) and two deep learning models (DeepSurv, NMTLR) to formulate prognostic models.

#### 2.4.1 Ensemble learning models

The Random Survival Forest (RSF) approach combines random forests with survival analysis techniques in order to address right-truncated data. It introduces new survival splitting rules for survival tree growth and innovative algorithms for estimating missing data. RSF also incorporates the event retention principle of survival forests, using it to define the overall mortality rate—a simple, interpretable measure that can be used as a predictor. RSF computations can be performed using the randomforestSRC (rfsrc) package.

Gradient Boosting with Component Linear Models (GLMboost) is a regression and classification algorithm based on gradient boosting tree, with GLMboost as its base model. Using an incremental gradient boosting approach, GLMboost improves the predictive power of the base model systematically while reducing its complexity through regularisation. One of GLMboost’s unique strengths is its adaptability to a wide range of data types, including categorical and continuous variables. Furthermore, it demonstrates the ability to address the common challenge of missing data, which is frequently encountered in datasets representing realistic virtual world scenarios.

Decision tree (dt) predict the value of the dependent variable by deriving simple decision rules from the available data. By contrast, boosting tree are an integrated learning approach that involves combining predictions from multiple models to improve overall performance. While decision tree partition the data by selecting the most appropriate features, boosting tree combiners combine multiple models to achieve superior generalisation performance.

Boosting tree (bt) is an integrated learning method that generates multiple weak learners iteratively and combines them into strong learners to improve prediction performance. Unlike decision tree, which are constructed independently, boosting tree are trained iteratively. Each round of training focuses on the samples that were incorrectly predicted in the previous round to improve model accuracy by gradually reducing bias. In survival analysis, boosting tree optimise the model parameters by minimising the loss function (e.g., negative log-likelihood), making them particularly suitable for dealing with nonlinear relationships and high-dimensional data.

#### 2.4.2 Deep learning models

DeepSurv is a survival analysis model based on deep learning that combines the traditional Cox proportional hazards model with deep neural networks to automatically identify complex nonlinear interactions between features. DeepSurv’s advantage over traditional methods lies in its ability to handle high-dimensional sparse data and capture complex relationships between features. The model estimates parameters by maximising a partial log-likelihood function; the hidden layer of the neural network learns a richer representation of features than traditional methods do.

Neural multi-task logistic regression (NMTLR) is a neural network model designed for survival analysis that can handle the survival prediction task at multiple time points simultaneously, improving prediction accuracy by sharing underlying feature representations and learning correlations between different time points. It employs a multi-task learning framework where each task corresponds to a specific time point and predicts the probability of an event occurring before that time point through logistic regression.

### 2.5 Model testing and evaluation

The performance of seven different models was assessed using various metrics, including the C-index, the combined cumulative/dynamic area under the curve (AUC) and the combined Brier score. The C-index indicates the discriminatory power of a survival model, with values greater than 0.7 suggesting usefulness. Higher C-index values were associated with a greater likelihood of model predictive success. Similarly, the AUC calculated from the ROC curve is a comparable metric to the C-index. Additionally, the Brier score assesses the model’s predictive accuracy, with values below 0.25 indicating practical application. Improved predictive accuracy was associated with lower Brier scores. Furthermore, Cox-Snell residual plots are graphical tools that can be used to assess the model’s goodness of fit.

### 2.6 Statistical analysis and model interpretability

LASSO regression was used to screen for survival risk factors, and univariate and multivariate Cox regression analyses were performed in SPSS 26.0 to identify independent prognostic factors (statistical significance was set at P < 0.05). Based on the screened features, a Cox proportional hazards regression model (CoxPH) and six machine learning (ML) models were constructed, including Random Survival Forest (RSF), component-wise linear Gradient Boosting (GLMboost), Decision Tree (dt), Boosting Tree (bt), Deep Survival Network (DeepSurv), and Neural Multi-Task Logistic Regression (NMTLR).

To comprehensively and accurately evaluate model performance, in addition to clinically recognized metrics—including the C-index (for assessing discriminative ability), integrated cumulative/dynamic AUC (for evaluating discriminative performance at different time points), and integrated Brier score (for measuring calibration between predicted probabilities and actual outcomes)—two goodness-of-fit metrics suitable for survival analysis were additionally incorporated: the Kolmogorov-Smirnov (KS) test and Cramer-von Mises (CvM) test. The KS test quantifies the overall discrepancy between the predicted and observed survival distributions by calculating the maximum distance between the two distributions. In contrast, the CvM test measures the average discrepancy between the distributions via an integral form and is more sensitive to deviations in the distribution tails. Together, these two metrics complement each other to more comprehensively validate the consistency between model predictions and clinical real-world data.

For the optimal model selected, in-depth interpretability analyses were conducted to clarify its internal working mechanism and enhance clinical utility:

Time-dependent variable importance analysis was used to identify key features that significantly influence survival outcomes at different follow-up time points, revealing the temporal dynamic patterns of variable effects; Partial correlation survival plots were generated to visualize the relationship curves between core features and survival probabilities, intuitively demonstrating the independent impact of a single feature on prognosis after controlling for other variables; SurvSHAP (an extended SHAP method dedicated to survival analysis) was employed to quantify the contribution of each feature to model predictions and clarify the direction of the association between feature values and risk predictions (either increasing or decreasing risk).

The construction of all ML models, performance evaluation (including calculation of the C-index, integrated AUC, integrated Brier score, KS test, and CvM test), and interpretability analyses were implemented using Python 3.12.9. The analysis relied on the following toolkits: data processing (Pandas, NumPy, SciPy), model construction (Scikit-learn, Lifelines, Scikit-survival, PyTorch, Torchvision), interpretability analysis (SHAP), and visualization (Matplotlib, Seaborn).

## 3 Results

### 3.1 Demographic and clinicopathological characteristics

A comprehensive cohort of 5,702 patients diagnosed with oesophageal cancer between 2010 and 2017 was carefully selected from the SEER database using pre-determined inclusion and exclusion criteria. Of these patients, 3,991 were assigned to the training set and the remaining 1,711 constituted the validation set. A further 300 patients treated at the First People’s Hospital of Kashi between 2015 and 2020 formed the external validation set. Statistical analysis using the chi-square test showed that there was no significant difference between the training set, the validation set, and the external validation set. Table 1 describes the clinicopathological characteristics of the three sets.

**TABLE 1:** General clinical data of training set and validation set samples of esophageal cancer patients in SEER Database [*n* (%)].

Characteristic	Training set (*n* = 3991) [n (%)]	Validation set (*n* = 1711) [n (%)]	External validation set (*n* = 300) [n (%)]	Χ^2^	*P*
Grade				3.566	0.735
G1	225 (5.6)	80 (4.7)	12 (4)		
G2	1772 (44.4)	764 (44.7)	140 (46.7)		
G3	1941 (48.6)	844 (49.3)	144 (48)		
G4	53 (1.3)	23 (1.3)	4 (1.3)		
Histologic type				1.609	0.952
ADC	2755 (69)	1200 (70.1)	204 (68)		
SCN	972 (24.4)	407 (23.8)	75 (25)		
EN	80 (2)	31 (1.8)	5 (1.7)		
CMSN	184 (4.6)	73 (4.3)	16 (5.3)		
Primari site				1.412	0.842
Lower third	3181 (79.7)	1355 (79.2)	231 (77)		
Middle third	622 (15.6)	274 (16)	54 (18)		
Upper third	188 (4.7)	82 (4.8)	15 (5)		
Stage				1.882	0.758
Localized	764 (19.1)	349 (20.4)	59 (19.7)		
Regional	1697 (42.5)	705 (41.2)	121 (40.3)		
Distant	1530 (38.3)	657 (38.4)	120 (40)		
Tstage				9.046	0.171
T1	1017 (25.5)	416 (24.3)	59 (19.7)		
T2	562 (14.1)	252 (14.7)	52 (17.3)		
T3	1941 (48.6)	818 (47.8)	155 (51.7)		
T4	471 (11.8)	225 (13.2)	34 (11.3)		
Nstage				3.468	0.748
N0	1284 (32.2)	555 (32.4)	95 (31.7)		
N1	1885 (47.2)	807 (47.2)	132 (44)		
N2	609 (15.3)	251 (14.7)	52 (17.3)		
N3	213 (5.3)	98 (5.7)	21 (7)		
Mstage				0.227	0.893
M0	2729 (68.4)	1162 (67.9)	202 (67.3)		
M1	1262 (31.6)	549 (32.1)	98 (32.7)		
Bone Metastasis				1.240	0.538
No	3704 (92.8)	1574 (92)	279 (93)		
Yes	287 (7.2)	137 (8)	21 (7)		
Lung Metastasis				0.342	0.843
No	3684 (92.3)	1573 (91.9)	275 (91.7)		
Yes	307 (7.7)	138 (8.1)	25 (8.3)		
Liver Metastasis				0.438	0.803
No	3437 (86.1)	1480 (86.5)	262 (87.3)		
Yes	554 (13.9)	231 (13.5)	38 (12.7)		
Radiothearepy				0.098	0.952
Yes	2706 (67.8)	1162 (67.9)	201 (67)		
No	1285 (32.2)	549 (32.1)	99 (33)		
Chemotherapy				0.736	0.692
Yes	905 (22.7)	405 (23.7)	67 (22.3)		
No	3086 (77.3)	1306 (76.3)	233 (77.7)		
Sex				0.474	0.789
Male	3251 (81.5)	1383 (80.8)	241 (80.3)		
Female	740 (18.5)	328 (19.2)	59 (19.7)		
Age				5.240	0.513
<60	680 (17.0)	292 (17.1)	41 (13.7)		
61–70	1361 (34.1)	552 (32.3)	98 (32.7)		
71–80	1294 (32.4)	572 (33.4)	109 (36.3)		
>81	656 (16.4)	295 (17.2)	52 (17.3)		

### 3.2 Analysis of prognostic factors of esophageal cancer

**FIGURE 1:**
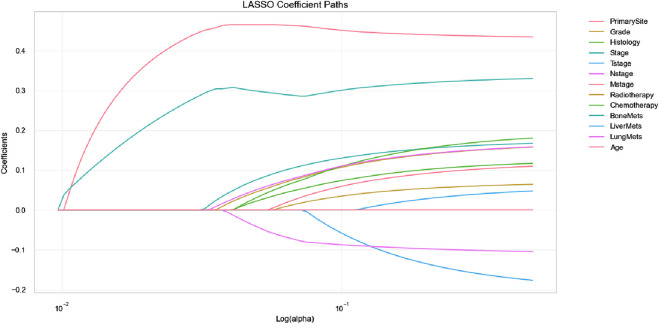
Feature selection using LASSO regression (The LASSO coefficient profiles depict the representation of 14 variables).

A total of 13 clinical parameters were included. LASSO regression analysis was used to screen for parameters with a p-value of less than 0.05, and 11 variables were retained (see Table 2; Figure 1). These 11 variables were then included in the Cox regression analysis, which identified Grade, Stage, Tstage, Nstage, Mstage, Radiotherapy, Chemotherapy, Bone metastasis, Liver metastasis, Lung metastasis and Age, along with 11 other variables with p-values below 0.05, as independent risk determinants of OS in oesophageal cancer (Table 3).

**TABLE 2:** LASSO variable screening results Table.

Variable	Coefficient	*Z* score	*P*
Mstage	0.416	11.627	<0.001
Stage	0.378	8.629	<0.001
Chemotherapy	−0.206	−4.987	<0.001
BoneMets	0.204	6.409	<0.001
Tstage	0.161	6.232	<0.001
Nstage	0.149	6.014	<0.001
LiverMets	0.146	3.777	<0.001
Grade	0.126	5.902	<0.001
Radiotherapy	−0.098	−4.486	<0.001
Age	0.054	4.799	<0.001
Histology	0.034	1.959	0.051
LungMets	0.030	2.081	0.037
Sex	0	−0.830	0.406
PrimarySite	0	−0.191	0.848

**TABLE 3:** Univariate and multivariate analysis of prognostic factors related to OS in patients with esophageal cancer.

Characteristic	Univariate analysis		Multivariate analysis	
	*HR* (95%*CI*)	*P*	*HR* (95%*CI*)	*P*
Tumor grade				
G1	Reference value		Reference value	
G2	2.632 (2.087,3.320)	<0.001	2.284 (1.807,2.886)	<0.001
G3	3.979 (3.159,5.012)	<0.001	2.833 (2.241,3.580)	<0.001
G4	4.315 (2.959,6.291)	<0.001	2.308 (1.575,3.381)	<0.001
Tumor stage				
Localized	Reference value		Reference value	
Regional	2.181 (1.913,2.486)	<0.001	1.878 (1.557,2.266)	<0.001
Distant	8.579 (7.536,9.765)	<0.001	2.504 (1.995,3.144)	<0.001
TNM-T				
T1	Reference value		Reference value	
T2	1.269 (1.112,1.448)	<0.001	1.216 (1.056,1.399)	0.007
T3	1.652 (1.497,1.824)	<0.001	1.225 (1.088,1.380)	<0.001
T4	4.391 (3.864,4.989)	<0.001	1.957 (1.700,2.253)	<0.001
TNM-N				
N0	Reference value		Reference value	
N1	2.534 (2.307,2.784)	<0.001	1.487 (1.322,1.674)	<0.001
N2	2.588 (2.297,2.917)	<0.001	1.556 (1.352,1.791)	<0.001
N3	4.612 (3.924,5.42)	<0.001	2.192 (1.832,2.623)	<0.001
TNM-M				
M0	Reference value		Reference value	
M1	6.652 (6.139,7.207)	<0.001	2.735 (2.338,3.198)	<0.001
Bone Metastasis				
No	Reference value		Reference value	
Yes	6.524 (5.727,7.432)	<0.001	1.938 (1.692,2.220)	<0.001
Lung Metastasis				
No	Reference value		Reference value	
Yes	4.916 (4.344,5.563)	<0.001	1.301 (1.140,1.486)	<0.001
Liver Metastasis				
No	Reference value		Reference value	
Yes	5.214 (4.724,5.754)	<0.001	1.379 (1.228,1.547)	<0.001
Radiothearepy				
No	Reference value		Reference value	
Yes	0.567 (0.521,0.616)	<0.001	0.794 (0.720,0.876)	<0.001
Chemotherapy				
No	Reference value		Reference value	
Yes	1.307 (1.188,1.437)	<0.001	0.591 (0.527,0.663)	<0.001
Age(Y)				
<60	Reference value		Reference value	
61–70	0.965 (0.78,1.195)	0.745	1.079 (0.963,1.210)	0.190
71–80	1.001 (0.809,1.24)	0.992	1.116 (0.995,1.251)	0.061
>81	1.343 (1.078,1.674)	0.009	1.546 (1.357,1.761)	<0.001

### 3.3 Model comparison

**FIGURE 2:**
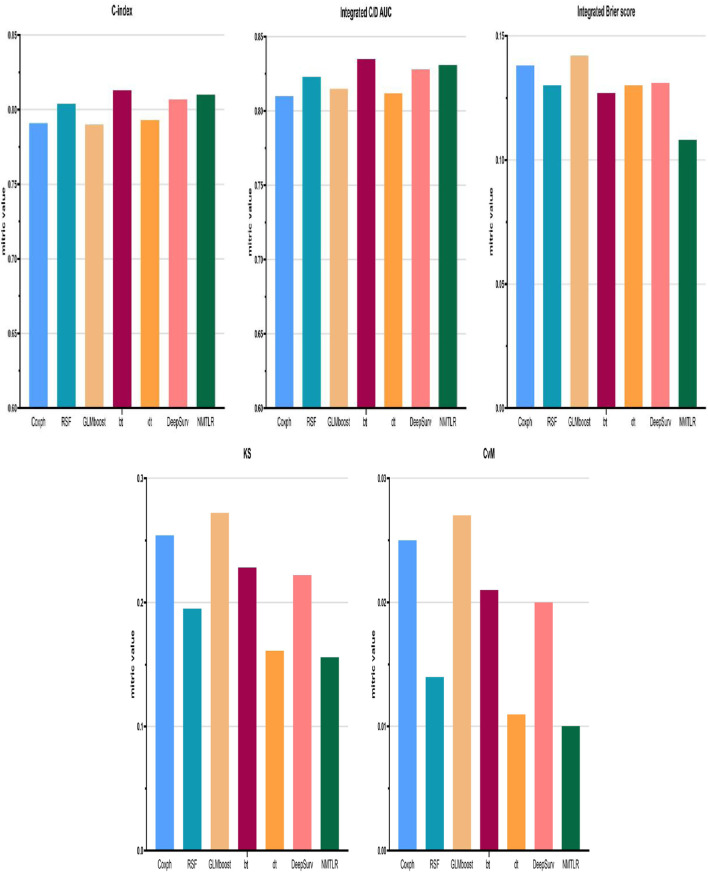
Model performance for the training set was displayed in the form of bar plots. Cox proportional hazard (Coxph); random survival forest (rfsrc); gradient boosting with component-wise linear (glmboost); boosting tree (bt); decision tree (dt); Probabilistic Survival Prediction with Deep Neural Networks (DeepSurv); neural multi-task logistic regression (NMTLR).

**FIGURE 3:**
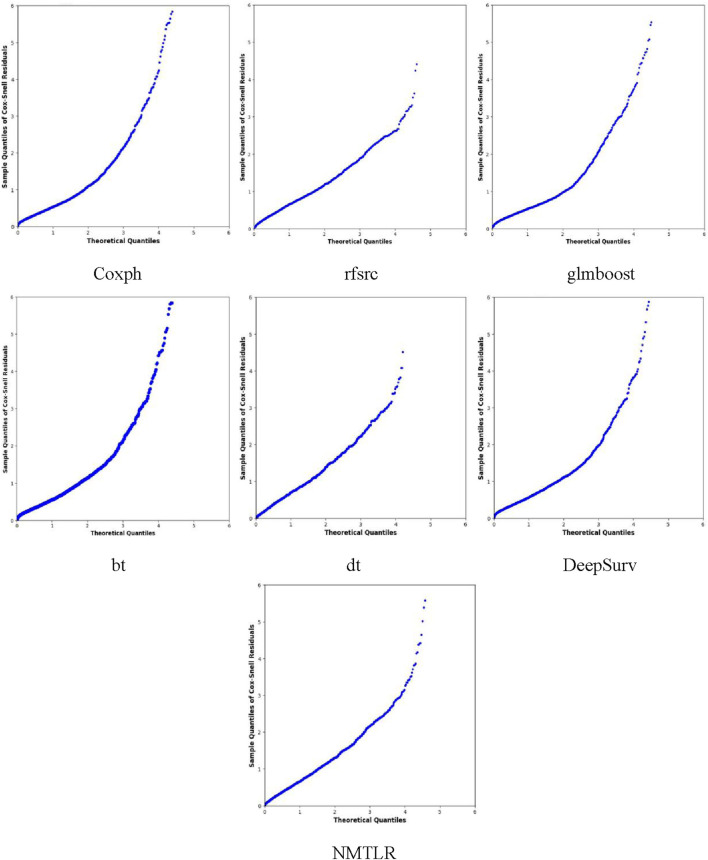
The Cox-Snell residual plots were displayed for all models in the training dataset.

We developed seven machine learning (ML) prediction models and evaluated their performance using internal (training and validation sets) and external validation cohorts, with results summarized in Table 4 and Figures 2, 3 (supplementary results for validation and external validation sets are shown in Supplementary Figures S1, S2). To address the potential interpretive complexity of Cox-Snell residual plots (Figure 3), we additionally incorporated Kolmogorov-Smirnov (KS) and Cramer-von Mises (CvM) metrics—two statistical measures that quantify the goodness-of-fit between predicted and observed survival distributions—for more intuitive performance comparison.

**TABLE 4:** The all models’ performance in the training set, validation set, and external validation set.

Prediction model name	C-index	Integrated C/D AUC	Integrated brier score	KS	CvM
Training set					
Coxph	0.791	0.810	0.138	0.254	0.025
RSF	0.804	0.823	0.130	0.195	0.014
GLMboost	0.790	0.815	0.142	0.272	0.027
Boosting tree	0.813	0.835	0.127	0.228	0.021
Decision tree	0.793	0.812	0.130	0.161	0.011
DeepSurv	0.807	0.828	0.131	0.222	0.020
NMTLR	0.810	0.831	0.108	0.156	0.010
Validation set					
Coxph	0.794	0.821	0.136	0.251	0.025
RSF	0.808	0.838	0.129	0.200	0.015
GLMboost	0.796	0.831	0.140	0.276	0.028
Boosting tree	0.808	0.841	0.130	0.225	0.022
Decision tree	0.796	0.824	0.129	0.175	0.011
DeepSurv	0.810	0.842	0.129	0.228	0.021
NMTLR	0.806	0.843	0.110	0.170	0.011
External validation set					
Coxph	0.775	0.788	0.142	0.256	0.022
RSF	0.783	0.801	0.136	0.179	0.013
GLMboost	0.774	0.789	0.145	0.255	0.024
Boosting tree	0.786	0.804	0.138	0.231	0.019
Decision tree	0.764	0.782	0.141	0.172	0.013
DeepSurv	0.783	0.808	0.139	0.226	0.019
NMTLR	0.774	0.791	0.129	0.169	0.011

Model performance metrics (Table 4) demonstrated that all models achieved favorable predictive performance across datasets, with integrated Brier scores below 0.15. For the training set, the NMTLR model stood out with the lowest integrated Brier score (0.108) and competitive values in C-index (0.810) and integrated C/D AUC (0.831); in the validation set, it maintained the lowest integrated Brier score (0.110) and the highest integrated C/D AUC (0.843); even in the external validation set, it exhibited stable performance (integrated Brier score = 0.129, C-index = 0.774). Regarding KS and CvM: lower CvM values indicate closer alignment between predicted and observed survival.The NMTLR model has the lowest CvM values (training set: 0.010; validation set: 0.011; external validation set: 0.011) and KS values (training set: 0.156; validation set: 0.170; external validation set: 0.169) in all datasets, further supporting the model’s discriminative ability. NMTLR exhibits consistent performance across different cohorts.

Figure 2 presents the performance comparison of all models in the training set using bar plots, with the x-axis listing model types [Cox proportional hazard (Coxph), random survival forest (rfsrc), gradient boosting with component-wise linear (glmboost), boosting tree (bt), decision tree (dt), probabilistic survival prediction with deep neural networks (DeepSurv), and neural multi-task logistic regression (NMTLR)] and the y-axis representing key metrics (C-index, integrated C/D AUC, integrated Brier score, KS, CvM). As shown, the NMTLR model has the highest integrated C/D AUC and the lowest integrated Brier score and CvM in the training set—visually validating its optimal balance of discriminative ability and goodness-of-fit.

Figure 3 displays the Cox-Snell residual plots for all models in the training set, where the x-axis represents theoretical quantiles (expected residual values under a well-fitted model) and the y-axis represents observed Cox-Snell residuals. The low CvM values (Table 4) complement these plots, confirming that all models still have acceptable goodness-of-fit to the training data—with the NMTLR model’s minimal CvM further reinforcing its reliability. The Cox-Snell residual plots for validation and external validation sets (Supplementary Figure S2) show similar trends, and their corresponding CvM values (Table 4) support the models’ cross-cohort stability. Comprehensively considering all metrics (including KS/CvM for intuitive goodness-of-fit assessment), visualizations, and cross-dataset consistency, the NMTLR model exhibits the most balanced and superior performance, making it the optimal choice for predicting esophageal cancer patients’ overall survival.

**FIGURE 4:**
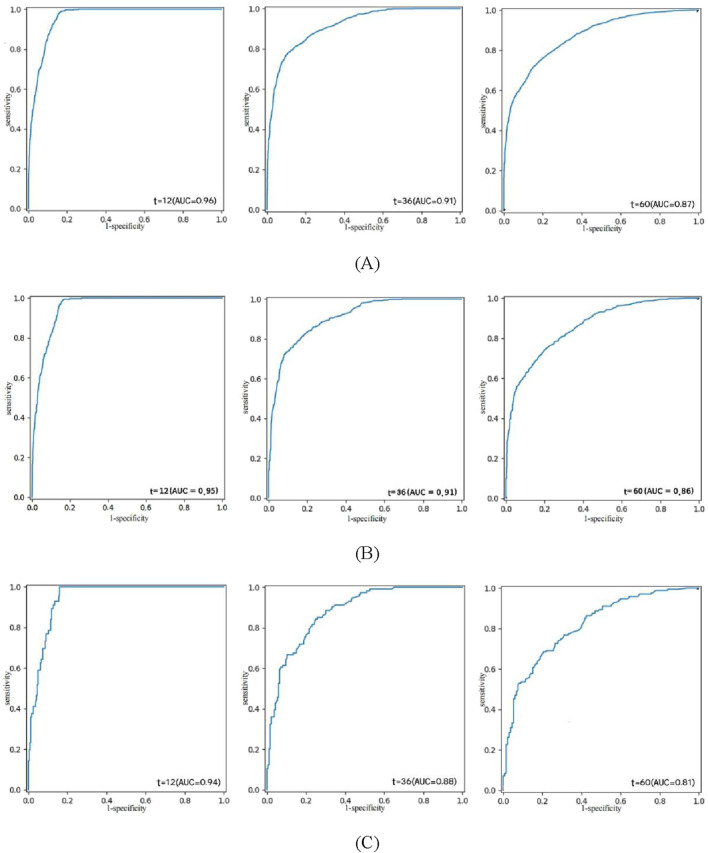
ROC curve analysis of the NMTLR model was used to evaluate the accuracy of the 1-, 3-, and 5-year predictions. **(A)** training set. **(B)** validation set. **(C)** external validation set.

The composite Brier scores, composite C/D AUC, and C-index for the NMTLR model in the training, validation, and external validation sets were 0.109, 0.112, and 0.128; 0.831, 0.839, and 0.794; and 0.808, 0.807, and 0.779, respectively.The ROC curves (Figure 4) illustrate that at 1, 3, and 5 years the subjects’ The areas under the operational eigenvalues are as follows: 0.956, 0.915, and 0.868 for the training set; 0.953, 0.910, and 0.866 for the validation set; and 0.948, 0.891, and 0.830 in the external validation set, respectively.

### 3.4 Model interpretability

To further evaluate the optimal model, we performed a global analysis to gain a comprehensive understanding of its performance.

#### 3.4.1 Time-dependent feature importance

**FIGURE 5:**
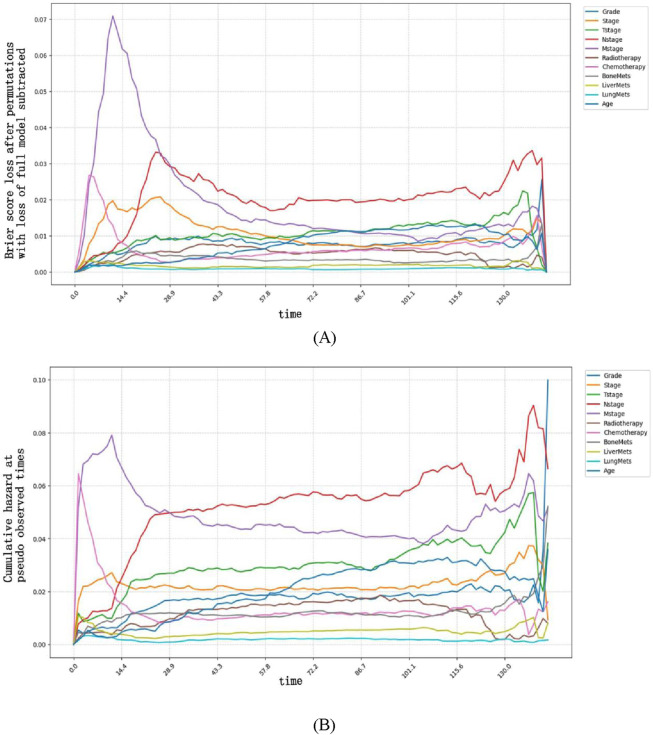
Time-dependent feature importance for the training set. **(A)** The Brier score loss after permutation; **(B)** the C/D AUC loss after permutation. The y-axis represents the variation in the loss function after permuting each covariate.

In our study, we examined the impact of each variable on the model’s global predictions. Since each variable may have a different impact at different time points, we quantified the time-dependent importance of variables in the NMTLR model by measuring how much the model’s predictive performance degrades when a specific variable is replaced with random noise (while keeping other variables unchanged). We used two metrics to assess this performance degradation: the increase in Brier score and the decrease in time-dependent AUC after variable replacement (see Figure 5).

Variable importance exhibited clear time-dependent patterns: a larger increase in Brier score (or larger decrease in AUC) after replacing a variable indicated that this variable had a more critical role in predicting OS at that specific time point. Specifically, the Brier score-based analysis revealed more distinct time-dependent patterns. Our findings indicate that M stage had the strongest impact on OS prediction when survival time was less than approximately 25 months, as replacing this variable resulted in the most significant degradation of model performance (i.e., the largest increase in Brier score and the greatest decrease in time-dependent AUC). In contrast, after 25 months, N stage became the most influential predictor, with its replacement leading to the most significant performance degradation.

These time-dependent patterns carry important clinical implications for personalized management of esophageal cancer patients:

For patients with a predicted survival time of <25 months (e.g., those with advanced M stage at diagnosis), the prominent role of M stage suggests that early intervention targeting metastatic lesions—such as systemic therapy or local ablative treatments for oligometastases—may be prioritized to improve short-term survival. Close monitoring of metastatic progression (e.g., regular imaging assessments) in this cohort could also help adjust treatment strategies promptly.

For patients surviving beyond 25 months, the dominant influence of N stage indicates that lymph node status remains a key prognostic driver in the mid-to-long term. This supports the clinical value of thorough lymph node evaluation (e.g., via endoscopic ultrasound or PET-CT) even in the later follow-up period, as persistent or recurrent nodal disease may require aggressive salvage therapy to extend survival.

Together, these findings highlight that prognostic factors for esophageal cancer vary dynamically over time, emphasizing the need for time-stratified risk assessment and adaptive treatment planning in clinical practice.

#### 3.4.2 Partial dependence survival profiles

**FIGURE 6:**
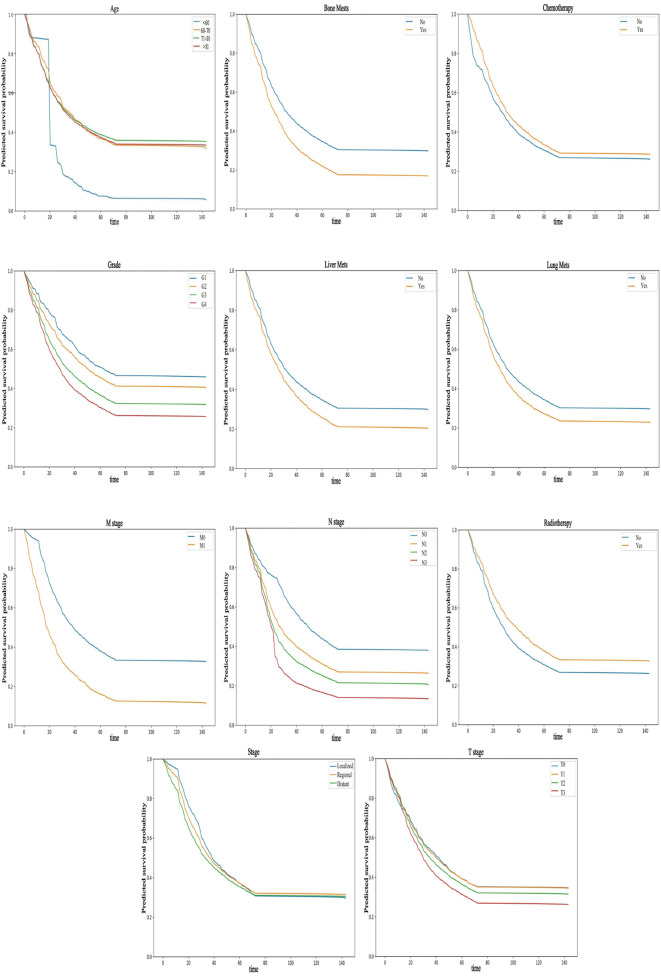
PDP can provide a global explanation for the NMTLR model. The survival function values of any covariates are depicted on the y-axis. The larger the differences between levels of a factor, the greater the impact of the same factor on OS. A lower numerical value indicates a lower probability of survival.

Partially dependent survival profiles (PDPs) can also be used to provide an overall explanation of the NMTLR model (see Figure 6). PDPs illustrate how the predicted survival probability (i.e., the probability of patients surviving for a given length of time) of the entire cohort changes relative to survival time when only one determinant is changed, while holding all the other determinants constant in the training dataset. In Figure 6, the y-coordinate represents the predicted survival probability (ranging from 0 to 1), which reflects the model’s estimated probability that a patient survives beyond a specific time point (x-coordinate, in months). If the labelled bands (representing confidence intervals) are thin and nearly overlap, this indicates that the overall predicted survival probability remains similar regardless of the values of these variables. If the bands are wider and do not overlap, it suggests that even slight alterations in their values can result in substantial variations in predicted survival probability. For instance, changes in variables such as M stage, N stage, age, grade, bone metastasis, liver metastasis, lung metastasis, radiotherapy, etc., can significantly impact predicted survival probability. Additionally, the predicted survival probability declined more rapidly in M1 patients than in M0 patients. Similarly, N3 patients, patients under 60 years of age, G4 patients, patients with bone, liver or lung metastases, and patients who did not receive radiotherapy experienced a more rapid decline in predicted survival probability.

#### 3.4.3 Aggregated SurvSHAP values summary

SurvSHAP(t) is an extension of the SHAP (SHapley Additive exPlanations) framework tailored for survival analysis, designed to quantify the contribution of each feature to model predictions across time points (Dai et al., 2025). Built on the axiomatic foundation of Shapley values from game theory, SurvSHAP(t) assigns a time-dependent importance score to each feature by evaluating its marginal contribution to the prediction error when removed from all possible subsets of features (Wang et al., 2025). For survival models like NMTLR, this involves decomposing the predicted cumulative hazard function into individual feature contributions, such that the sum of all SurvSHAP(t) values for a patient approximates the difference between their predicted risk and the average risk of the entire cohort (Sato et al., 2024).

**FIGURE 7:**
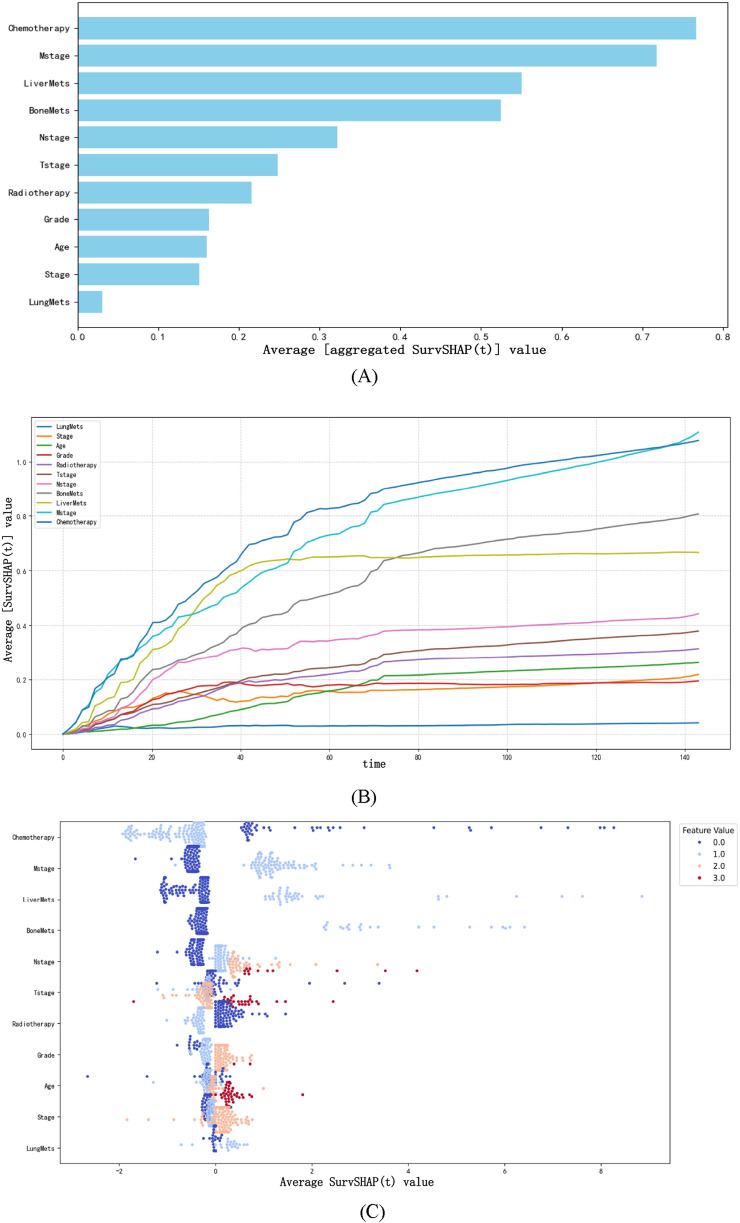
The SurvSHAP summary plot provides an overall interpretation of the global impact. **(A)** The length of the bar chart represents the overall significance of the variables. **(B)** The curve graph displays the cumulative importance of each variable. **(C)** Each point on the bee swarm plot represents a specific feature of a particular patient. The y-coordinate of the point is determined by the feature it represents, while the x-coordinate is determined by its impact on the model output. The features on the y-axis are sorted according to their significance.

We computed and illustrated the SurvSHAP summary plots for the NMTLR model, which includes 11 features ranked by their overall impact on OS. Figure 7A displays the global importance of variables, defined as the mean absolute SurvSHAP(t) value across all time points and observations. Figure 7B illustrates the temporal variability of each variable’s significance, with the y-axis representing the average absolute SurvSHAP(t) value at each time point, highlighting how feature importance fluctuates over the follow-up period.

In the bee swarm plot (Figure 7C), variables are ordered by their mean absolute SurvSHAP(t) value (descending) to reflect overall importance. Each point represents a single patient’s SurvSHAP(t) value for a specific variable: the x-axis indicates the magnitude and direction of the feature’s impact on OS prediction (positive values = increased mortality risk; negative values = reduced risk). The color gradient (coolwarm palette) encodes the original feature values (red = higher values; blue = lower values), not the SurvSHAP(t) magnitude. Among the 11 features, chemotherapy had the highest overall impact, followed by M stage, liver metastases, bone metastases, N stage, T stage, and radiotherapy.

## 4 Discussion

Esophageal cancer remains a globally prevalent malignancy with complex pathogenesis, posing substantial challenges to clinical management. Despite advances in diagnosis and treatment, its OS remains suboptimal, underscoring the urgent need for accurate prognostic models to guide personalized patient care (Morgan et al., 2022).

Our study addresses this gap by developing and validating interpretable ML models for OS prediction in esophageal cancer. Unlike previous studies that focused primarily on model performance (Xu et al., 2022; Nopour, 2024), we integrated LASSO and multivariate Cox regression to identify key risk factors and constructed 7 ML models, with NMTLR emerging as the superior performer. Its consistent accuracy across training, internal, and external validation sets (1-, 3-, 5-year AUC >0.81; integrated Brier score <0.175) not only confirms its robustness but also, more critically, offers actionable insights through interpretability analyses—transcending the “black box” limitations of traditional ML.

A key innovation of our study lies in the cross-regional validation strategy, utilizing data from the SEER database (primarily North American patients) and an external cohort from the First People’s Hospital of Kashi, China. The NMTLR model maintained high performance across these distinct populations (external validation AUC: 0.82–0.85), indicating its ability to generalize beyond geographical and demographic boundaries. This cross-cultural robustness suggests that the core prognostic factors identified (e.g., M stage, N stage, metastasis status) are universally critical, regardless of regional differences, while also implying that the model is resilient to variations in data collection practices or clinical workflows. Such generalizability is a prerequisite for the model’s potential as a global clinical tool, reducing the need for population-specific recalibration.

The 11 prognostic factors identified (grade, stage, T/N/M stages, radiotherapy, chemotherapy, bone/liver/lung metastases, age) align with clinical knowledge but are contextualized within a dynamic framework that enhances their practical utility. For example, while tumour stage is known to correlate with prognosis (Detterbeck et al., 2024), our time-dependent analyses reveal nuanced dynamics: M stage dominates early survival (<25 months), whereas N stage becomes critical later. This temporal stratification enables targeted clinical strategies—such as prioritizing systemic therapy for metastatic disease in the short term and intensifying lymph node surveillance in long-term survivors—that go beyond generic recommendations.

Our focus on interpretability—via time-dependent importance, partial correlation plots, and SurvSHAP—delivers unique clinical value. SurvSHAP, in particular, quantifies the direction and magnitude of each feature’s impact on OS prediction at the individual patient level. When paired with partial correlation survival curves (which visualize how survival probabilities shift with changes in these features), these insights help clinicians avoid unnecessary treatment-related toxicity in low-benefit subgroups and prioritize interventions for patients where the model indicates meaningful survival benefit—moving beyond descriptive prognostic associations to provide actionable, patient-tailored guidance that aligns with clinical decision-making needs.

Regarding the methodological strengths of our time-dependent analyses, we used permutation-based Brier score loss and C/D AUC loss to assess variable importance. The Brier score, as a proper scoring rule, quantifies the calibration and precision of predicted probabilities simultaneously, making it more sensitive to temporal shifts in predictive performance compared to AUC (which focuses on discrimination alone). This sensitivity explains why Brier score loss more clearly captured the transition of dominant factors from M stage (early) to N stage (late)—a phenomenon rooted in its ability to penalize both calibration errors (e.g., overestimating survival in M1 patients) and discrimination gaps across time points. This characteristic highlights the Brier score’s utility in survival model interpretation, as it aligns with clinical needs to trust both the order and magnitude of risk predictions.

Limitations of our study include its retrospective design and reliance on SEER and single-center external data. Future work will incorporate multi-center prospective cohorts across diverse regions to further validate the model’s generalizability. Additionally, exploring the impact of region-specific variables (e.g., dietary factors, local treatment guidelines) on model performance could provide deeper insights into optimizing predictions for specific populations. Enhancing visualization tools for real-time clinical use—such as interactive SurvSHAP dashboards—will further bridge the gap between ML outputs and bedside decision-making.

## 5 Conclusion

This study successfully constructed and validated an interpretable NMTLR model for predicting the OS of esophageal cancer patients, addressing key gaps in existing prognostic tools. By integrating data from the SEER database (primarily North American patients) and an external validation cohort from Kashi, China—paired with key risk factor screening via LASSO and multivariate Cox regression—the model exhibited robust predictive performance: across all datasets, 1-, 3-, and 5-year OS AUCs exceeded 0.81 and integrated Brier scores remained below 0.175, outperforming most previously reported models. Beyond accuracy, complementary interpretive tools (time-dependent variable importance analysis, partial correlation survival plots, and SurvSHAP plots) clarified the dynamic influence of factors (e.g., M stage dominating early survival <25 months, N stage critical in later periods), overcoming traditional machine learning’s “black box” limitation and transforming predictions into clinically actionable logic. This work provides clinicians with a tool to refine survival assessment and tailor treatment plans, while offering a framework for interpretable ML in oncology; limitations include retrospective design and lack of molecular data, and future efforts will expand multi-center prospective cohorts, integrate multi-omics data, and optimize interpretive tools to promote precision diagnosis/treatment and improve global patient outcomes.

## Data Availability

The raw data supporting the conclusions of this article will be made available by the authors, without undue reservation.
