# Supplementary material for: Interpretable machine learning models to predict survival in esophageal cancer: a study based on the SEER database and external validation in China
Source: Front Physiol. 2025 Oct 29;16:1665383. doi: 10.3389/fphys.2025.1665383 (PMC12605121; doi:10.3389/fphys.2025.1665383)
Supplement: Supplementary file 1 [file Supplementaryfile1.docx]

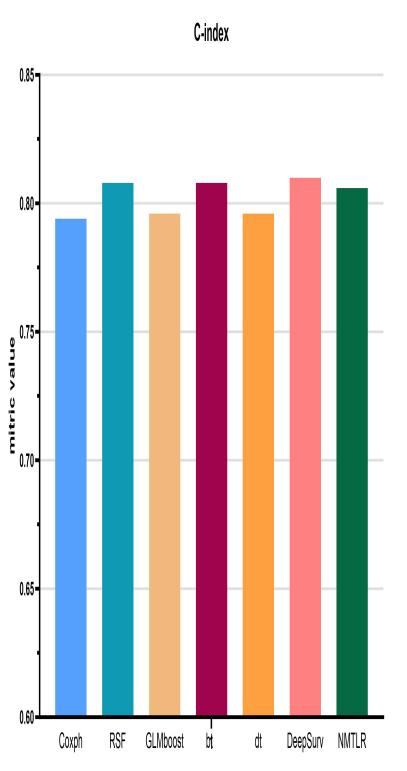

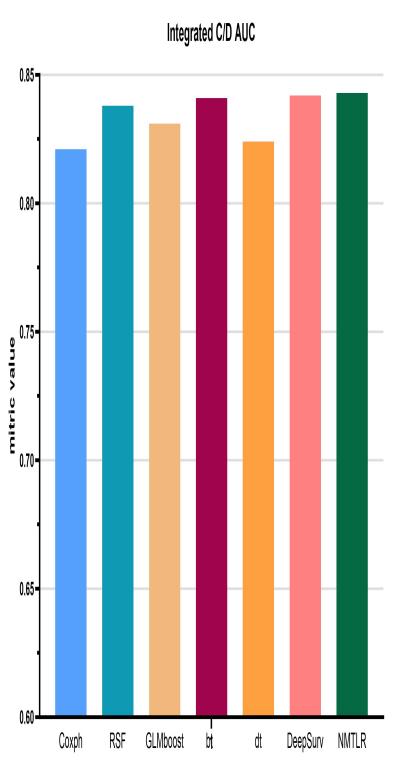

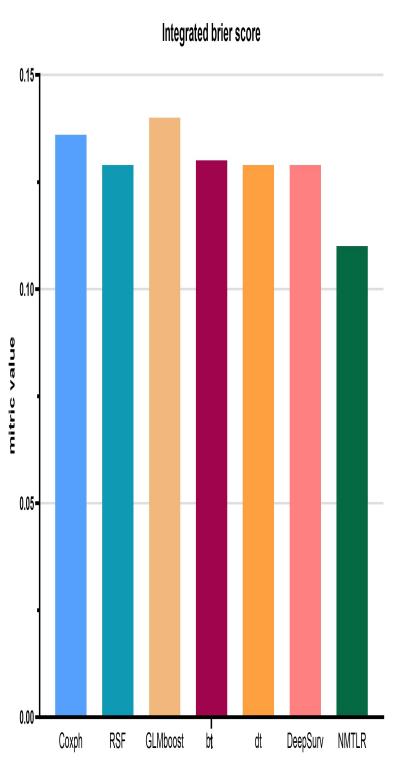


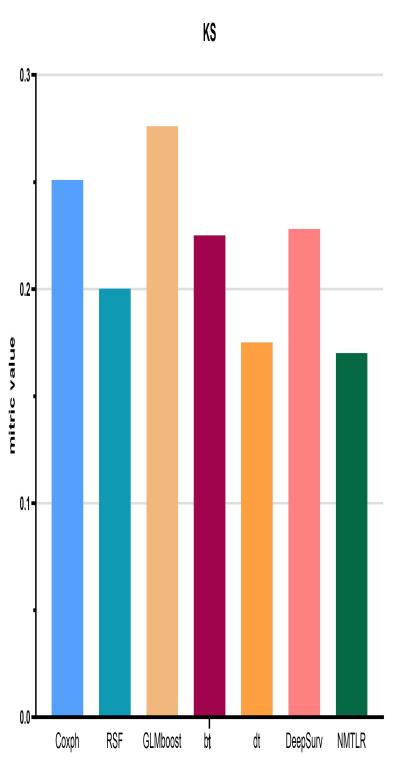

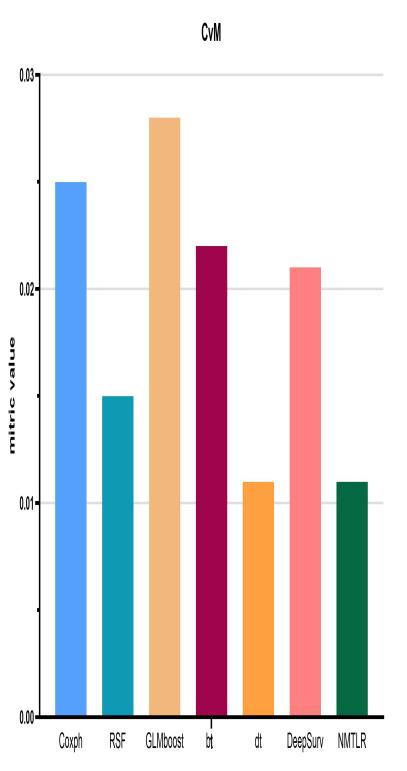


(A)


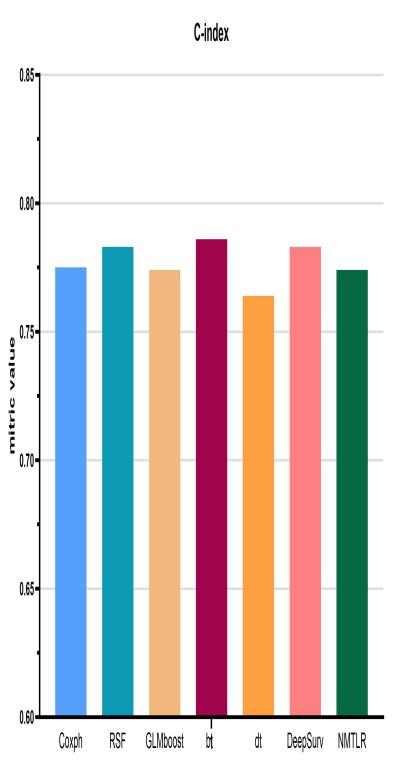

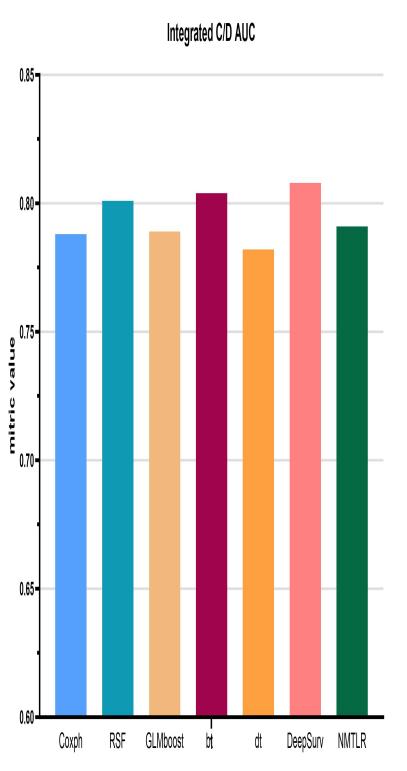

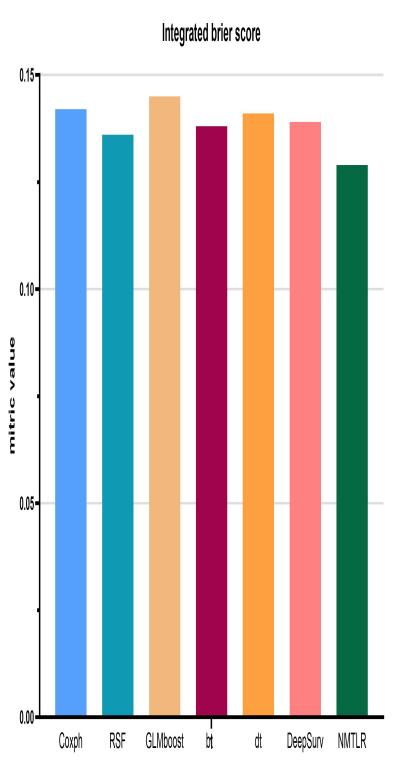


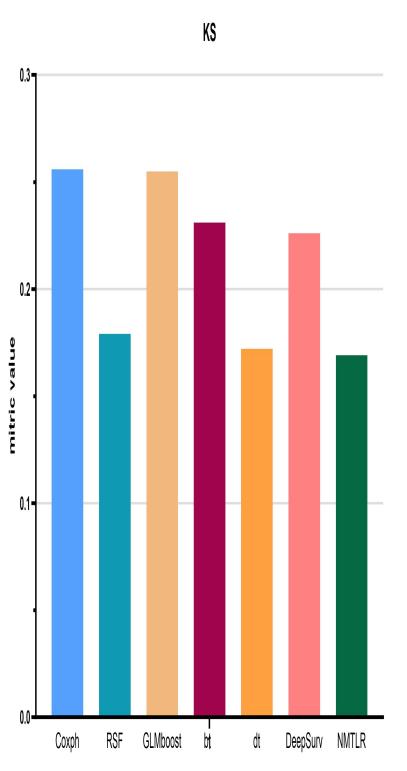

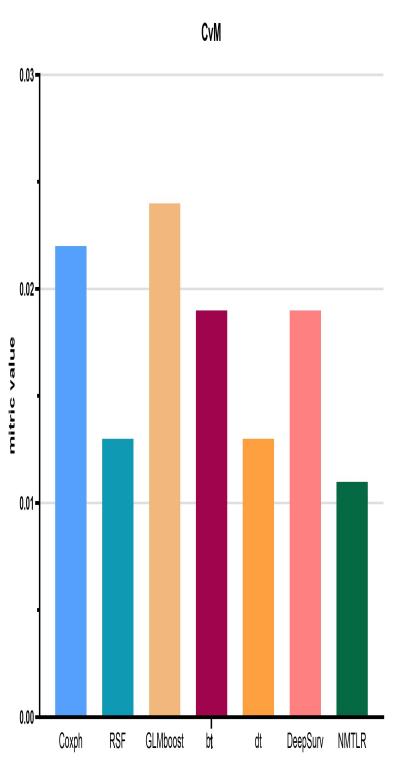


(B)

Figure S1 Model performance was displayed in the form of bar plots (A)Validation set; (B)External validation set


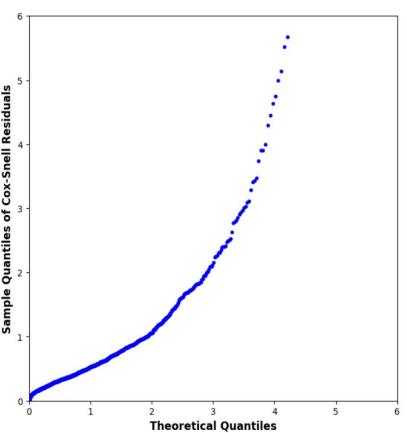

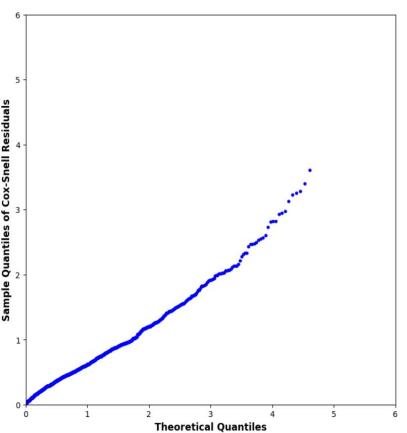

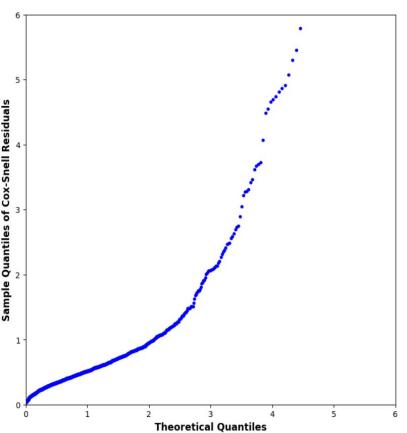


Coxph rfsrc glmboost


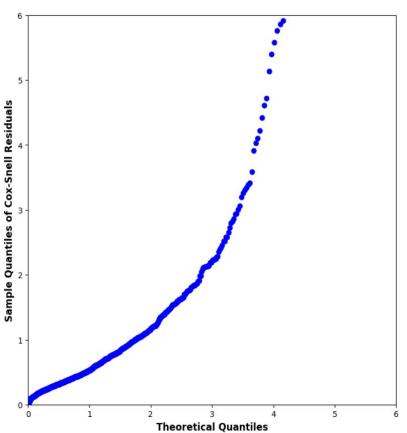

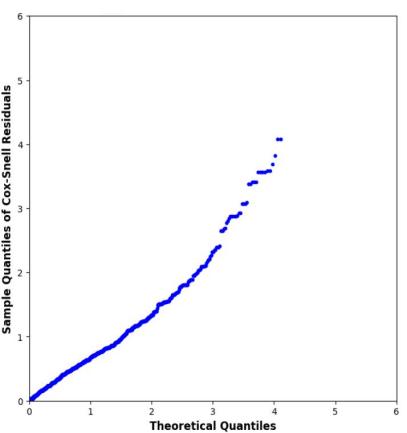

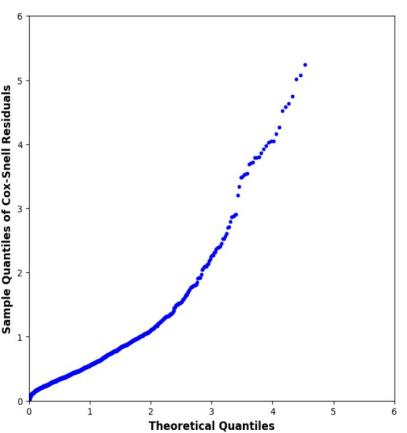


bt dt DeepSurv


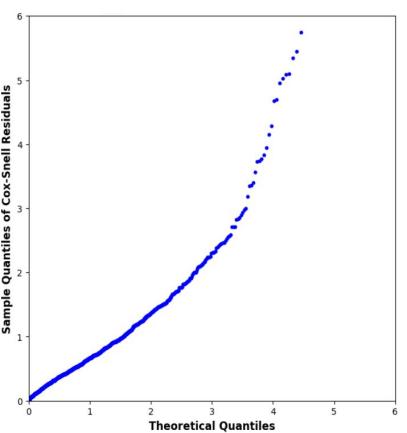


NMTLR

(A)


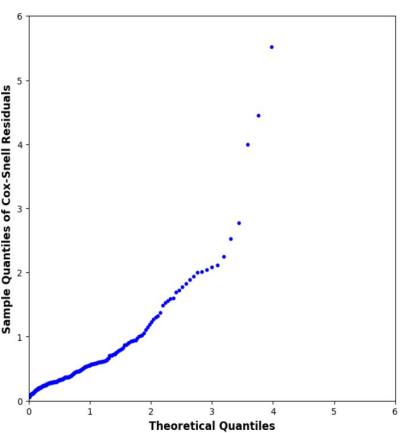

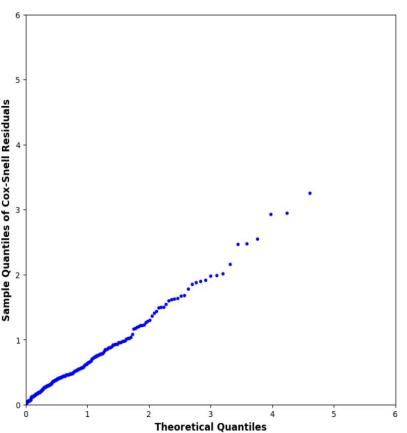

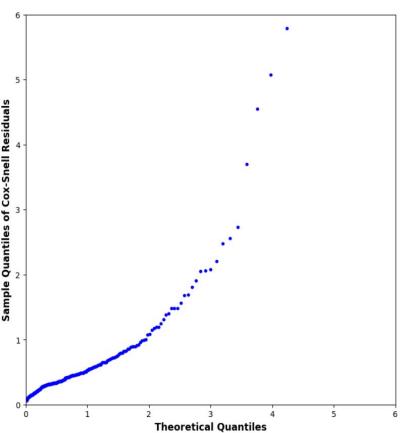


Coxph rfsrc glmboost


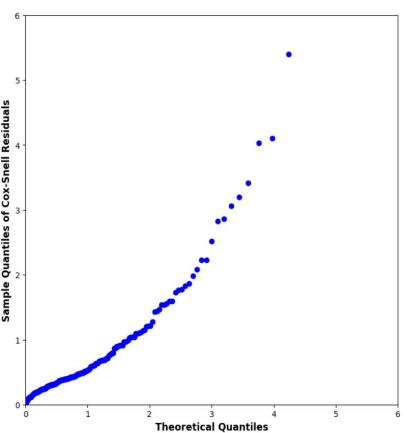

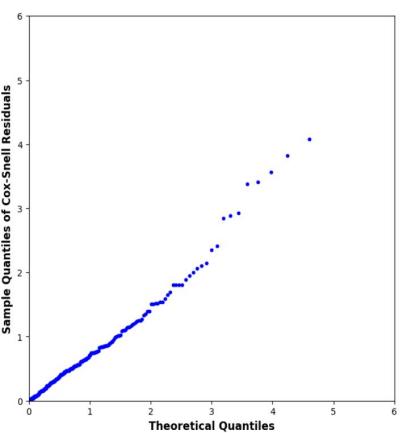

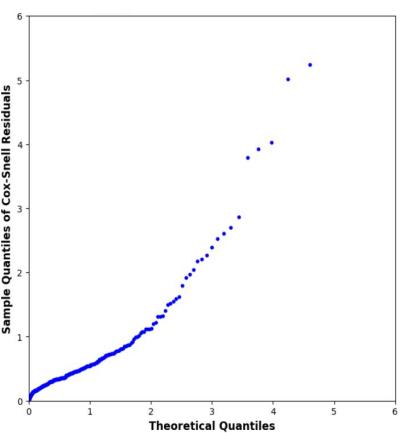


bt dt DeepSurv


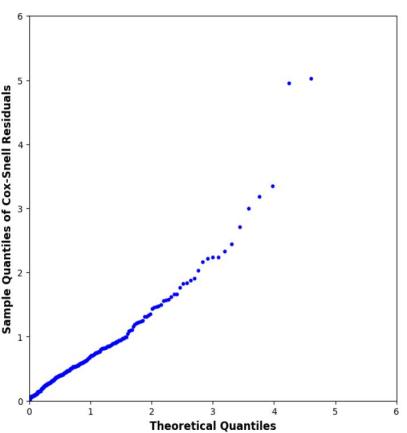


NMTLR

(B)

Figure S2 The Cox-Snell residual plots were displayed for all models (A)validation set; (B)external validation set


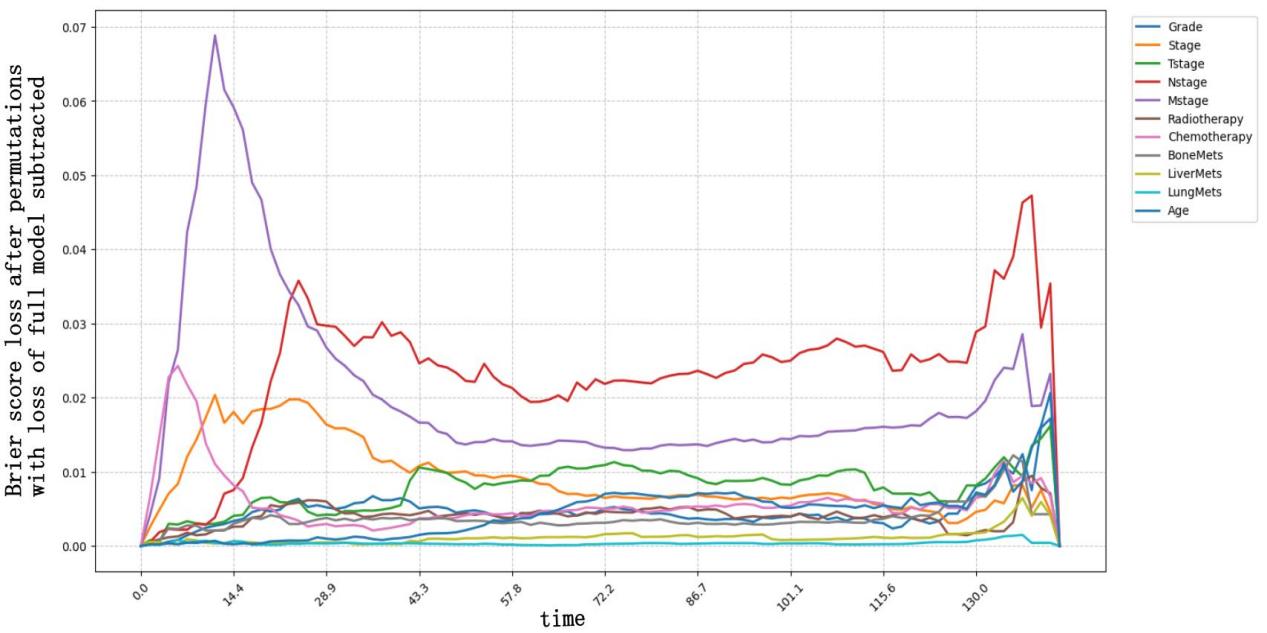


(A)


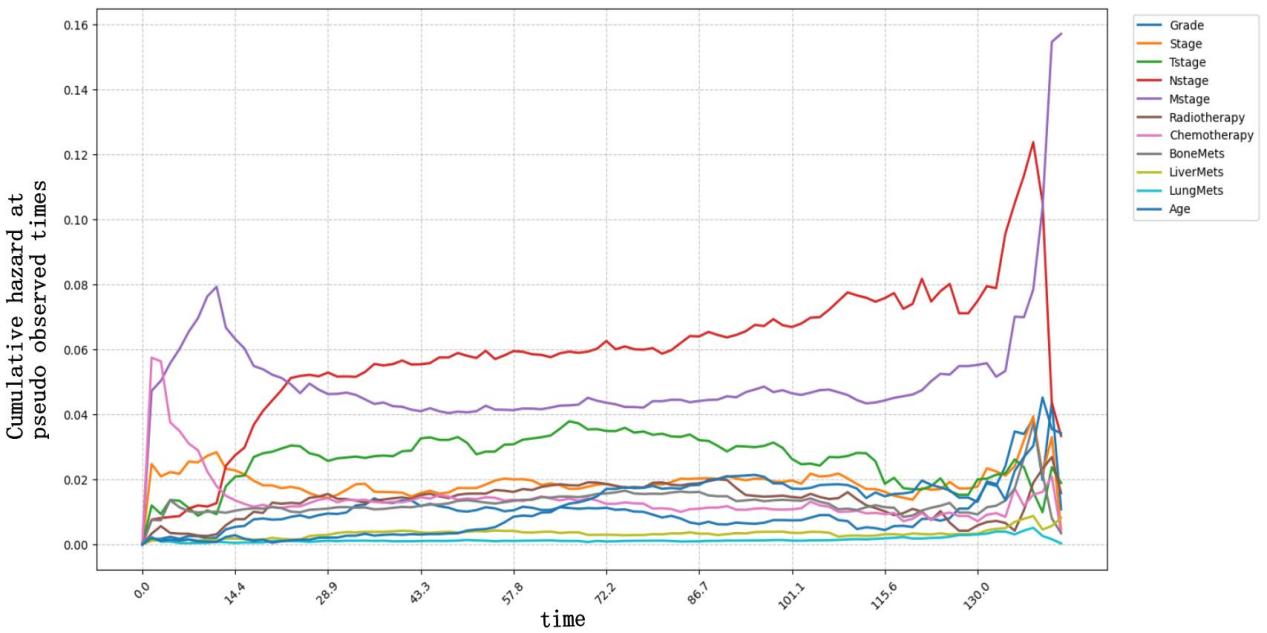


(B)


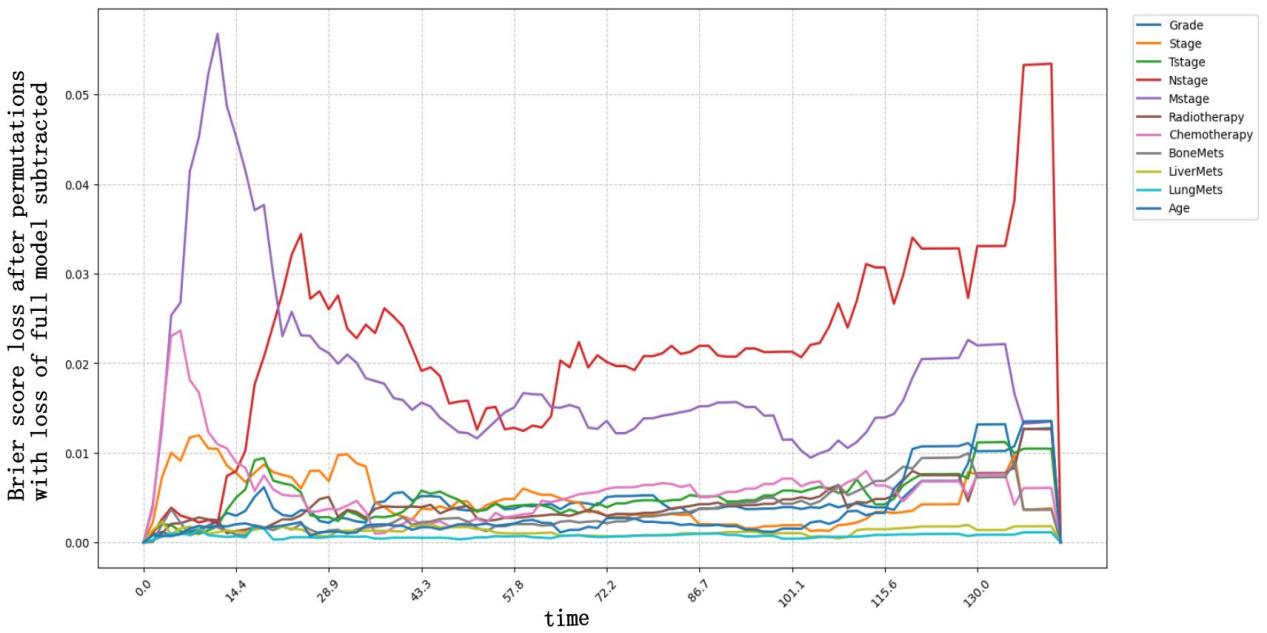


(C)


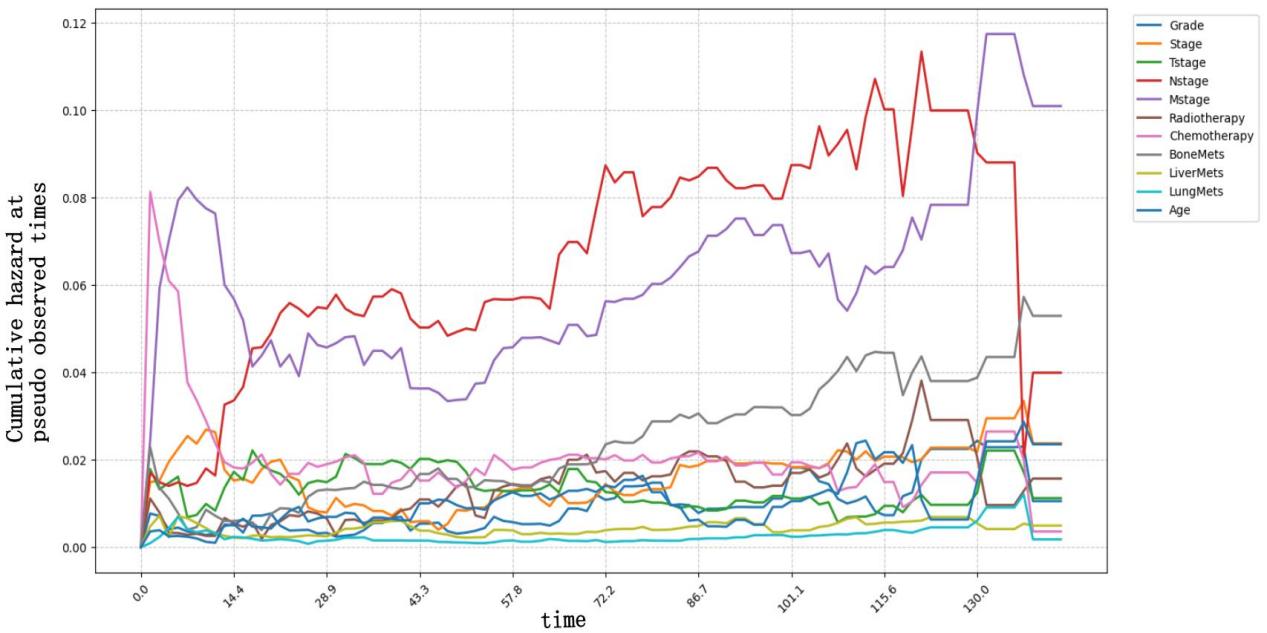


(D)

Figure S3 Time-­dependent feature importance, (A) The Brier score loss after permutation for the validation set; (B) the C/D AUC loss after permutation for the validation set; (C) The Brier score loss after permutation for the external validation set; (D) the C/D AUC loss after permutation for the external validation set
